# Using the embryo-uterus statistical model to predict pregnancy chances by using cleavage stage morphokinetics and female age: two centre-specific prediction models and mutual validation

**DOI:** 10.1186/s12958-023-01076-8

**Published:** 2023-03-27

**Authors:** Eva S. van Marion, Esther B. Baart, Margarida Santos, Linette van Duijn, Evert J. P. van Santbrink, Régine P. M. Steegers-Theunissen, Joop S. E. Laven, Marinus J. C. Eijkemans

**Affiliations:** 1 Division of Reproductive Endocrinology and Infertility, Department of Obstetrics and Gynaecology, Erasmus MC, University Medical Centre, PO Box 2040, 3000 CA Rotterdam, the Netherlands; 2 Department of Developmental Biology, Erasmus MC, University Medical Centre, PO Box 2040, 3000 CA Rotterdam, the Netherlands; 3 Fertility Center, Reinier de Graaf Hospital, Fonteynenburghlaan 5, 2275 CX Voorburg, the Netherlands; 4 Department of Obstetrics and Gynaecology, Erasmus MC, University Medical Centre, PO Box 2040, 3000 CA Rotterdam, the Netherlands; 5 Department of Data Science and Biostatistics, University Medical Centre, Utrecht University, PO Box 85500, 3508 GA Utrecht, the Netherlands

**Keywords:** Embryo transfer, In vitro fertilisation, Prediction model, Statistical models, Time-lapse imaging

## Abstract

**Background:**

The predictive capability of time-lapse monitoring (TLM) selection algorithms is influenced by patient characteristics, type and quality of data included in the analysis and the used statistical methods. Previous studies excluded DET cycles of which only one embryo implanted, introducing bias into the data. Therefore, we wanted to develop a TLM prediction model that is able to predict pregnancy chances after both single- and double embryo transfer (SET and DET).

**Methods:**

This is a retrospective study of couples (*n* = 1770) undergoing an in vitro fertilization cycle at the Erasmus MC, University Medical Centre Rotterdam (clinic A) or the Reinier de Graaf Hospital (clinic B). This resulted in 2058 transferred embryos with time-lapse and pregnancy outcome information. For each dataset a prediction model was established by using the Embryo-Uterus statistical model with the number of gestational sacs as the outcome variable. This process was followed by cross-validation.

**Results:**

Prediction model A (based on data of clinic A) included female age, t3-t2 and t5-t4, and model B (clinic B) included female age, t2, t3-t2 and t5-t4. Internal validation showed overfitting of model A (calibration slope 0.765 and area under the curve (AUC) 0.60), and minor overfitting of model B (slope 0.915 and AUC 0.65). External validation showed that model A was capable of predicting pregnancy in the dataset of clinic B with an AUC of 0.65 (95% CI: 0.61–0.69; slope 1.223, 95% CI: 0.903–1.561). Model B was less accurate in predicting pregnancy in the dataset of clinic A (AUC 0.60, 95% CI: 0.56–0.65; slope 0.671, 95% CI: 0.422–0.939).

**Conclusion:**

Our study demonstrates a novel approach to the development of a TLM prediction model by applying the EU statistical model. With further development and validation in clinical practice, our prediction model approach can aid in embryo selection and decision making for SET or DET.

## Background

Selection of embryos with the highest implantation potential remains a challenge during in vitro fertilisation (IVF) treatment. Nowadays this is an even more important task because of the preference for single embryo transfer (SET) to decrease the risks of a twin pregnancy. Since the beginning of human IVF, morphological assessment and scoring of an available cohort of embryos has been the method of choice to select or deselect embryos for transfer [1, 2]. This method was refined and extended during the last decades, for example by adding the evaluation of pronuclear stage morphology [3, 4] or blastocyst stage morphology [5, 6]. To promote standardization of this process, a consensus on morphological criteria for embryo assessment was reached [7].

Over the past decade, time-lapse embryo culture is increasingly used as a semi-quantitative tool to research the timing of embryo development and the correlation with implantation. Yet, there is still insufficient good quality evidence for superiority of embryo selection based on morphokinetic parameters compared to selection based on morphology [8, 9]. Results of studies on the correlation between time-lapse morphokinetics and implantation were used to develop embryo selection algorithms. Studies with different sample sizes and statistical approaches have led to either centre-specific, multicentre or generally applicable models [10–18]. Not all studies performed external validation after developing their algorithm, and a decreased predictive capability was observed during external validation in some cases [19–22]. Possible explanations are heterogeneous patient populations, culture conditions and transfer policy. This underlines the importance of centre-specific models.

The predictive capability of time-lapse selection algorithms can be influenced by patient characteristics, type of data included in the analysis and the used statistical methods. Most of the earlier developed morphokinetic embryo selection algorithms did not include patient characteristics. However, female age is a patient characteristic known to be important for implantation success. Significantly different implantation rates were demonstrated in different female age groups of embryos with the same grading according to four previously published models [21]. Furthermore, another study found that female age affected the timing of the cleavage division to the 2- and the 4-cell stage [23]. Thus, female age can be a confounding factor in embryo selection algorithms. In addition, the importance of analysing patients, rather than embryos, as independent observations was already emphasized [24]. Second, in most studies, selection algorithm development was based on an optimal timing for the duration of an interval between two cell stages, resulting in the deselection of embryos out of range. However, consensus about the optimal timing and therefore the appropriate cut-off values is not yet reached. Time-consuming annotations are usually needed because of the inclusion of parameters up until the 8-cell stage, and this reduces clinical applicability. Finally, previously published studies excluded double embryo transfers (DETs) resulting in only one implanted embryo, because of a partial observability problem. This results in a substantial loss of data and introduces selection bias. This particular group of patients cannot be analysed using a classical multilevel statistical model. The previously developed embryo-uterus statistical model (EU model) is able to overcome this problem by combining couple or cycle level effects with embryo level effects. The framework of this model was introduced by Speirs and colleagues, and it was further developed over time [25]. Roberts demonstrated the clinical applicability of this model to real IVF data [26]. Correlations between embryos that implanted simultaneously are included in this model [25, 27–30]. This provides the opportunity to analyse all SETs and DETs resulting in a twin- or no pregnancy, but importantly also DETs resulting in a singleton pregnancy.

Our aim was to develop a TLM prediction model that is able to predict pregnancy chances after SET and DET by using both morphokinetic parameters and female age. To this end, we used the EU statistical model, enabling the inclusion of cycles with DET that resulted in only one implanted embryo and thereby minimizing selection bias. We developed two models based on centre-specific data from two different clinics. After developing the models, they were cross validated to test if these centre-specific prediction models also show comparable performance in the other independent IVF clinic. In addition, we examined if the models can predict the chance of a twin pregnancy after DET and thereby aid in the decision between SET and DET.

## Methods

### Study population

A retrospective cohort included couples undergoing IVF with or without intracytoplasmic sperm injection (ICSI) at the Erasmus MC, University Medical Centre Rotterdam (clinic A) between January 2012 and June 2019, and at the Reinier de Graaf Hospital (clinic B), between January 2013 and June 2019. Embryos were cultured in an EmbryoScope time-lapse incubator (Vitrolife, Göteborg, Sweden). All couples during the study period were included, with the exception of couples without fertilized oocytes (as indicated by the presence of two pronuclei) or if their embryo(s) had not reached the 5-cell stage three days after fertilization. In addition, only cycles with autologous, fresh oocytes were included. From couples undergoing multiple cycles during the study period, only data from their first available treatment cycle was included.

### Ovarian stimulation, oocyte insemination, embryo culture and transfer

Women underwent routine ovarian stimulation by either a GnRH-agonist or -antagonist co-treatment protocol with recombinant- or urinary- follicle stimulating hormone (FSH; Menopur, Ferring, St. Prex, Switzerland, Gonal-F, Merck Serono, Switzerland, Bemfola, Gedeon Richter Benelux, Belgium, or Rekovelle, Ferring, St. Prex, Switzerland) [31]. Human recombinant chorionic gonadotropin (hCG) (Ovitrelle, Merck Serono, Switzerland, Pregnyl, Organon, the Netherlands) was used as a trigger of final follicular maturation. Oocytes were fertilized according to routine IVF or ICSI procedures. Inseminated and injected oocytes were placed in EmbryoSlide culture dishes (Vitrolife, Sweden) and cultured in an EmbryoScope time-lapse incubator (Vitrolife, Sweden). In clinic A, the culture medium used was G-1 PLUS (Vitrolife, Sweden) cleavage stage culture medium between January 2012 and November 2014 or SAGE 1-step (Origio/Cooper Surgical, Trumbull, CT, USA) culture medium between November 2014 and June 2019 at 36.8 degrees Celsius, 7% oxygen and 5–6% carbon dioxide (Table 1). In clinic B the culture medium used was either Sage CM (Origio/Cooper Surgical, USA) culture medium between January 2013 and May 2015, SAGE 1-step (Origio/Cooper Surgical, USA) or G-TL (Vitrolife, Sweden) culture medium between June 2015 and October 2015 and SAGE 1-step (Origio/Cooper Surgical, USA) culture medium between October 2015 and June 2019 at 36.9 degrees Celsius, atmospheric oxygen and 5–6% carbon dioxide (Table 1). Embryo transfer was performed on day 3 and embryos were cryopreserved on day 4 or day 5 until the 1^st^ of April 2019. Afterwards, due to a change in laboratory policy at clinic A, embryo transfer selection was performed on day 5 after fertilization. This concerned 24 treatment cycles included in this cohort. In both clinics, it is standard care to transfer a single embryo. Only women aged 38 years or older without medical contra-indications or women undergoing their third or higher treatment cycle can opt for double embryo transfer. Embryo selection for transfer was not aided by time-lapse information and was performed on a single image acquired by the EmbryoScope at 66–68 h post-fertilization or -injection. Embryo morphology was ranked according to the number of blastomeres, fragmentation, equality of blastomere size and cell contact. Top ranking embryos contained eight blastomeres of equal size, with less than 10% fragmentation and maximum cell contact between the blastomeres. Additionally, if embryos with comparable quality were present on day 3 at clinic B, the number of blastomeres on day 2 (44 ± 1 h post insemination/injections) was added to the selection criteria followed by early cleavage according to previous literature to prefer early cleavage above late cleavage [32–34]. The number of gestational sacs was confirmed by ultrasound at 12 weeks of gestation.

**Table 1:** Patient- and treatment characteristics of cycles included in the time-lapse analysis

		**Clinic A**	**Clinic B**
**Couples**		706	1064
**Number of analyzed embryos (fresh transfer)**		784	1274
**Female age** (years)		34.3 (30.4–38.6)	35.5 (31.8–39.0)
**Fertilization method**	**IVF**	233 (33%)	107 (10.0%)
	**ICSI**	473 (67%)	959 (90.0%)
**Culture medium**	**Sage 1**	479 (67.8%)	544 (51.1%)
	**Sage CM**	0 (0%)	491 (46.1%)
	**Vitrolife**	228 (32.2%)	29 (2.7%)
**Oxygen**		7%	atmospheric
**Carbon dioxide**		5–6%	5–6%
**Temperature (°C)**		36.8	36.9

Each cycle is derived from a unique patient couple. Data are presented as number (%) or median (IQR)

*Abbreviations*: *IVF *in vitro fertilization, *ICSI* Intracytoplasmic sperm injection

### Time-lapse monitoring and assessment

Images were recorded automatically in seven focal planes with 15 μm intervals, every 10–15 min. The EmbryoScope uses a monochrome CCD camera with a single red LED at 635 nm with an exposure time of < 0.1 s per image, and a total light exposure time < 50 s per day per embryo. For IVF, t = 0 in both clinics was defined as the time of insemination. At clinic A t = 0 for ICSI was defined as the time of injection of the last oocyte, with the whole procedure taking between 20–50 min depending on the number of oocytes. Clinic B defined t = 0 for ICSI as the time halfway through injecting the spermatozoa into the available oocytes. Manual annotations were performed by four specifically trained members of our team according to the definitions and guidelines by Ciray and colleagues [35]. We annotated the time of PN appearance (tPNa), number of pronuclei, the first frame where both pronuclei were faded (tPNf), as well as the exact timing of reaching the 2, 3, 4, 5, 6, 7- and 8-cell stage (t2, t3, t4, t5, t6, t7 and t8). All four trained members of our team annotated the same set of ten embryos, and we tested the inter-observer agreement by calculating intra-class correlation coefficients (ICC). We found excellent agreement (intra-class correlation coefficient (ICC) > 0.9) for tPNa, tPNf and the cleavage divisions up until the 5-cell stage. Moderate agreement (ICC < 0.5) was found for the cleavage divisions between the 6- and the 8-cell stage.

The following time intervals between different developmental points were calculated: t3-t2 represents the interval between the 2-cell stage and the 3-cell stage and t5-t4 represents the interval between the 4-cell stage and the 5-cell stage.

### Statistical analysis

First, we selected the parameters to test for their predictive value when included into the model. As the TLM parameters up to the 5-cell stage showed the highest inter-observer agreement, we focused on these time points. Furthermore, to make the model more independent of fertilization method and culture conditions, we aimed to evaluate the duration of time intervals, rather than a specific time point of reaching a certain cell stage. We selected the duration of the interval between the 2- and the 3-cell stage (t3-t2), a parameter that was also previously shown to correlate with implantation [10, 17, 36]. In addition, the duration of the interval between the 4- and the 5-cell stage was selected, as this showed large variation and it does not overlap with t3-t2. We also added the time until the first cleavage division (t2), a parameter known to correlate with implantation [33, 37].

Using these parameters, we performed manual backward selection for inclusion in a multivariable EU-regression analysis. Predictors were eliminated from the model according to a relaxed criterion, a *p*-value > 0.3. We used a custom-written code in R [25–29]. The model consists of two logistic regression sub models, the ‘E’ and the ‘U’ part. Where the ‘E’ part describes the chance that in the case of a suitable recipient an embryo will develop and the ‘U’ part the chance that the recipient is suitable. In this way, the correlation between two transferred embryos is taken into account. This gives the opportunity to analyse all SETs, DETs resulting in a twin- or no pregnancy, but also DETs resulting in a singleton pregnancy. The number of gestational sacs was used as the outcome variable. For adequate performance of the model, the outcome variable should be close to implantation to avoid interference of other possible confounders. The model is fitted using direct maximization of the likelihood. Variations on the restricted cubic splines degrees of freedom (df) were considered; we started with two df for all variables and adjusted this when necessary.

After developing the models, we evaluated the predictive performance of both models. Discrimination, as expressed the area under curve (AUC), displays the ability of our models to correctly differentiate between women with a pregnancy and women without a pregnancy after embryo transfer during IVF/ICSI treatment [38]. Perfect discrimination is indicated by an AUC of 1 whereas no discrimination is indicated by an AUC of 0.5. The AUC was calculated using the method suggested by Harrell et al. [39]. The degree of agreement between predicted probabilities and observed outcomes is called calibration [38]. In our models, this will be the predicted probability of a pregnancy and the observed pregnancy rate. We assessed calibration graphically. In the case of perfect calibration, the plot shows a diagonal line with a slope of 1 and an intercept of 0.

Internal validation of both models on the prediction of pregnancy following SET was performed via bootstrapping. The prediction model was built on each bootstrap sample. The average optimisms, the differences between the calibration slope and AUC of the bootstrap prediction model on the entire dataset and of the bootstrap sample, were calculated and the apparent calibration slope and AUC were corrected accordingly. Most of the already existing TLM selection models did not include female age. To better understand the performance of our prediction models in the field of TLM models, we also provided an AUC of internal and external validation without female age.

## Results

### Patient- and treatment characteristics

The morphokinetic data from 706 IVF or IVF-ICSI treatment cycles performed at clinic A, resulted in 784 transferred embryos that were used to develop prediction model A. In 628 cycles SET was performed and in 78 DET. For the development of prediction model B, morphokinetic data from 1064 IVF or IVF-ICSI cycles performed at clinic B, resulting in 1274 transferred embryos were used. In 854 cycles SET was performed and in 210 DET. Female age, fertilization method and culture characteristics of the cycles included in this study are shown (Table 1). Outcomes, in terms of the number of implanted embryos, are also shown (Table 2).

**Table 2:** Pregnancy outcomes of cycles included in the time-lapse analysis. The number of gestational sacs was determined by ultrasound at 12 weeks of gestation

	**Clinic A**			**Clinic B**		
	**Not pregnant**	**One gestational sac**	**Two gestational sacs**	**Not pregnant**	**One gestational sac**	**Two gestational sacs**
**Single embryo transfer**	385 (54.5%)	243 (34.4%)	0 (0%)	536 (50.4%)	318 (29.9%)	0 (0%)
**Double embryo transfer**	53 (7.5%)	21 (3.0%)	4 (0.6%)	137 (12.9%)	55 (5.2%)	18 (1.7%)

Data are presented as number (%)

### Prediction model A (based on data from clinic A)

**Fig. 1:**
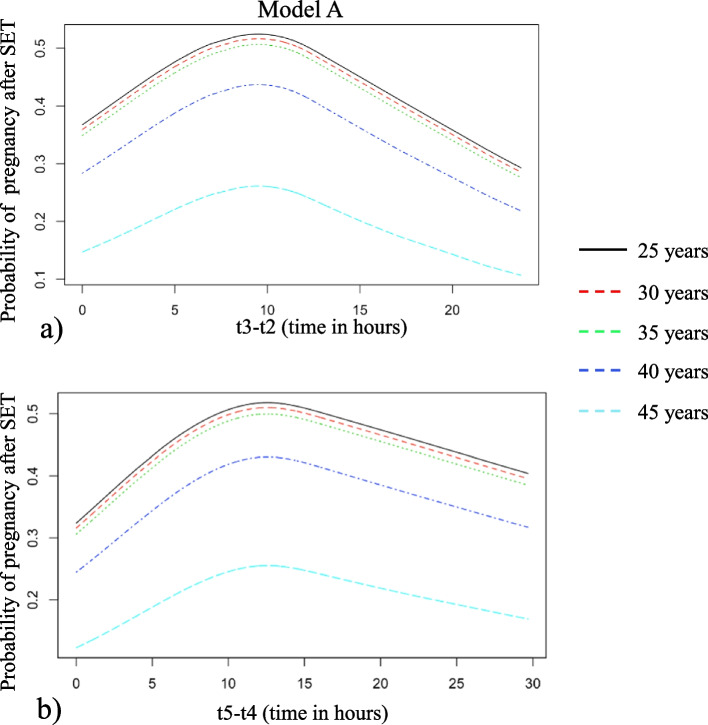
Prediction of pregnancy chances of model A after single embryo transfer using female age and (**a**) t3-t2, given that t5-t4 is 13 h, and (**b**) t5-t4 given that t3-t2 is 11 h. The different coloured lines depict different female ages

We performed manual backward selection in a multivariable EU-regression analysis with the following parameters: female age, t2, t3-t2 and t5-t4. As the relationship between these parameters and gestational sacs was non-linear, they were included using restricted cubic splines. This resulted in a final significant model including female age, t3-t2 and t5-t4, all with restricted cubic splines. The timing of fertilization can be different between normal IVF and IVF-ICSI, influencing the timing of subsequent morphokinetic events. This model, however, is independent of fertilization method, because t2 was excluded and only time intervals remained in the final model. These time intervals are not impacted by a delay in time to fertilization for IVF compared to ICSI. A visual representation of the calculations of pregnancy chances made by the model is given (Fig. 1). To depict the non-linear predictive effects of t3-t2 and t5-t4, two plots were made for each of these variables respectively, with separate curves for selected values of female age, keeping the other variable at a constant value close to the median (Fig. 1a and b). This is only done for the purpose of illustration; the model itself can predict pregnancy chances for all possible values of female age and the two morphokinetic parameters. Pregnancy chances after double embryo transfers can also be predicted. The optimal duration of t3-t2 is between 8 and 12 h. Pregnancy chances decline for all embryos that were slower or faster during this interval across all female ages. The optimal duration of t5-t4 is between 10 and 15 h.

### Prediction model B (based on data from clinic B)

**Fig. 2:**
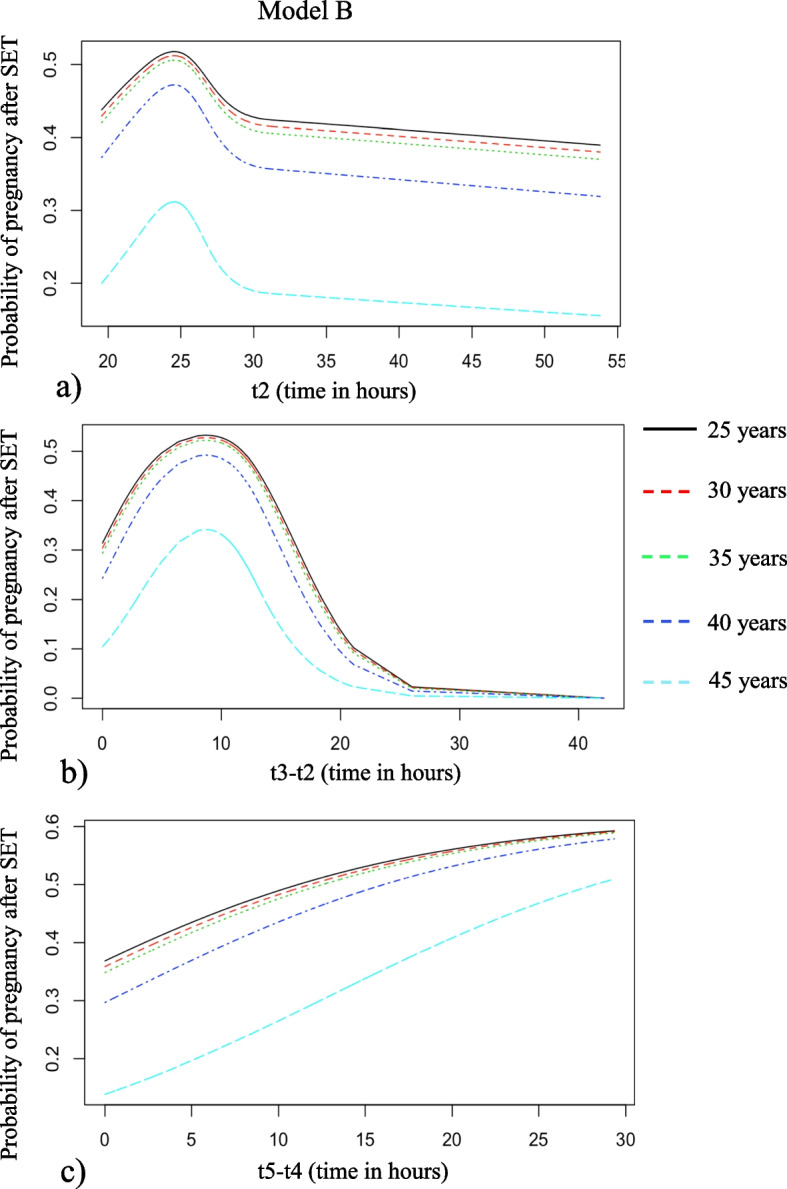
Prediction of pregnancy chances of model B after single embryo transfer using female age and (**a**) t2, given that t3-t2 is 11 h and t5-t4 is 13 h; **b** t3-t2 given that t2 is 25 h and t5-t4 is 13 h and (**c**) t5-t4 given that t2 is 25 h and t3-t2 is 11 h. The different coloured lines depict different female ages

Again, we performed manual backward selection in a multivariable EU-regression analysis with the following parameters: female age, t2, t3-t2 and t5-t4, using restricted cubic splines. This resulted in a final significant model including female age, t2, t3-t2 and t5-t4. Only t5-t4 remained in the final model as a linear variable, the other variables were included as restricted cubic splines. To depict the predictive effects of t2, t3-t2 and t5-t4 three plots were made for each of these variables respectively, with separate curves for selected values of female age, keeping the other variables at a constant value close to the median (Fig. 2a, b and c). Again, this is only done for the purpose of illustration. The optimal timing of t2 is between 23 and 27 h, and the optimal duration of t3-t2 is between 7 and 11 h. The pregnancy chance increases with an increasing duration of t5-t4.

### Inclusion of the variables to the Embryo or Uterus part of the model

Female age and the morphokinetic parameters were added to the ‘E’ part of both models. No covariates were added to the ‘U’ part of the model, therefore this part of the model was the same for all patients. We performed a likelihood ratio test to check whether the best fit was for the model with female age in the ‘E’ part compared to the model with female age in the ‘U’ part. The best-fitted model was the one with female age in the ‘E’ part according to AIC. Even without adding covariates to the ‘U-part’ of the model, this part still consists of an intercept. This intercept accounts for interdependence in DET transfers.

### Internal and external validation of model A

**Fig. 3:**
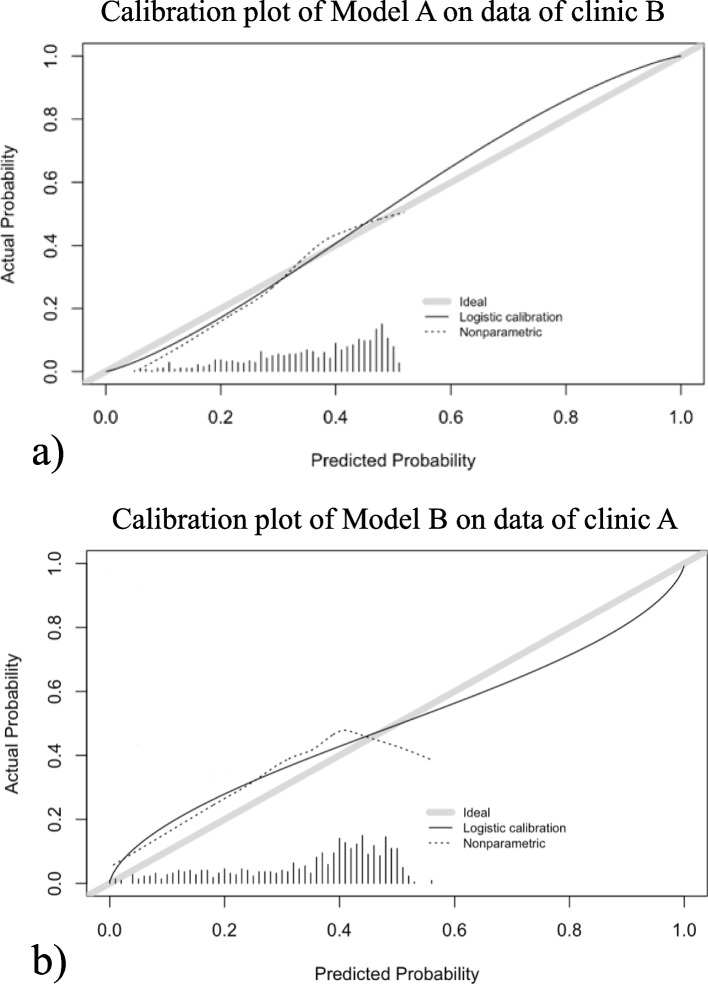
**a** Predicted probabilities by model B are plotted against the actual probability in the dataset of clinic A (solid black lines). **b** Predicted probabilities by model A are plotted against the actual probability in the dataset of clinic B (solid black line). The grey lines represent perfect calibration

We compared the observed percentage of pregnancies in the analysed dataset with the fitted percentage by the EU-model (Table 3). The smallest deviation of observed and fitted percentages by model A was 0.8% in the category SET and not pregnant or one implanted embryo. The largest deviation was 4.9% in the category DET and not pregnant. Internal validation of the prediction of pregnancy following SET via bootstrapping showed overfitting of model A with a calibration slope of 0.765 and an AUC of 0.60. The calibration plot of external validation of model A on data of clinic B is shown (Fig. 3a). The AUC was 0.65 (95% CI: 0.61–0.69) and the calibration plot showed a slope of 1.223 (95% CI: 0.903–1.561).

**Table 3:** Pregnancy outcome of the analysed cycles in terms of gestational sacs, as observed by ultrasound at 12 weeks of gestation. The observed percentages in the data and the fitted percentages by the prediction models are given

		**Model A**			**Model B**		
		**Not pregnant**	**One** **gestational sac**	**Two gestational sacs**	**Not pregnant**	**One gestational sac**	**Two gestational sacs**
**Single embryo transfer**	Observed	61.3%	38.7%	0%	62.8%	37.2%	0%
	Fitted	62.1%	37.9%	0%	63.4%	36.6%	0%
**Double embryo transfer**	Observed	67.9%	26.9%	5.1%	65.2%	26.2%	8.6%
	Fitted	63.0%	30.4%	6.6%	63.6%	25.6%	10.8%

Data are presented as number (%)

Internal validation of model A without female age, so only with the TLM parameters t3-t2 and t5-t4, resulted in an AUC of 0.57. External validation of this same model, on data of clinic B, showed an AUC of 0.58.

### Internal and external validation of model B

For model B the smallest deviation of observed and fitted percentages was 0.6% in the category SET and not pregnant or one implanted embryo. The largest deviation being 2.2% in the category DET and two implanted embryos (Table 3). Internal validation of the prediction of pregnancy following SET via bootstrapping showed minor overfitting of model B with a calibration slope of 0.915 and an AUC of 0.65. The calibration plot of external validation of model B on the data of clinic A is shown (Fig. 3b). The calibration plot showed evidence of underestimation of the pregnancy chance. The AUC was 0.60 (95% CI: 0.56–0.65) and the calibration slope 0.671 (95% CI: 0.422–0.939).

Internal validation of model B without female age, so only with the TLM parameters t2, t3-t2 and t5-t4, resulted in an AUC of 0.61. External validation of this same model, on data of clinic B, showed an AUC of 0.56.

### Applicability of the prediction models in the decision for SET or DET

**Fig. 4:**
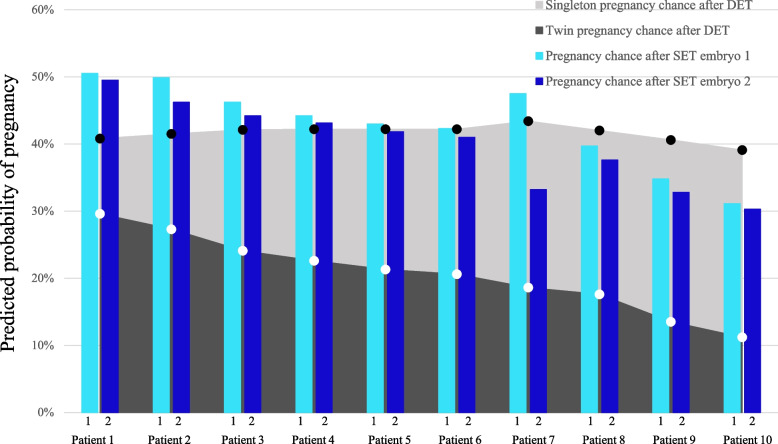
Illustration of the predicted probability of pregnancy after transfer of embryos originating from 10 patients of clinic A, where a double embryo transfer (DET) was performed. Patients were selected according to at least a 30% pregnancy chance predicted by our model A (according to morphokinetic parameters and female age), of both embryos. The light blue and dark blue bars represent the individual predicted probability of pregnancy after single embryo transfer (SET) for the first and second embryo. The white dots indicate the predicted probability of a twin pregnancy after transfer of both embryos originating from one patient; the black dots indicate the predicted probability of a singleton pregnancy after DET. Abbreviations: DET, double embryo transfer; SET, single embryo transfer

The decision for SET or DET is a consideration between the optimal chance of pregnancy and to avoid the risks of a twin pregnancy. We investigated if our prediction model could aid in this consideration. As an example we investigated predictions of embryos originating from 10 patients of clinic A where a DET was performed (Fig. 4). These patients were selected on the basis that both transferred embryos had at least a predicted pregnancy chance of 30% using model A (according to morphokinetic parameters and female age). The model can predict an individual chance of pregnancy for each embryo separately, but can also predict the chance of a singleton and twin pregnancy after DET. For example, both embryos originating from patient 5 give a 42–43% predicted pregnancy chance when transferring these embryos separately (SET) according to our model A. In addition, the model also predicts that when both embryos are transferred, the singleton pregnancy chance remains 42%, but with a risk for a twin pregnancy of 21%. So transferring the second embryo does not increase pregnancy success, but only constitutes a risk for twinning. On the other hand, for patient 10 both embryos give a pregnancy chance of 30–31% when transferred separately. After DET of both embryos originating from patient 10, the singleton pregnancy chance is 39%, with a twin pregnancy chance of 11%. In this case, DET would increase pregnancy success, but with an 11% risk of a twin pregnancy. Considering these predictions before embryo transfer are helpful in the decision between SET and DET.

## Discussion

Here, we aimed to develop a TLM prediction model that is able to predict pregnancy chances after SET and DET. This work resulted in two centre-specific prediction models that predict the chance for achieving a pregnancy based on a limited number of morphokinetic parameters and female age. Our used methods are new in the field and add another perspective to handling the data generated by time-lapse incubators and how this information could be useful for decision making.

First, using the EU statistical model enabled us to include both SET and DET cycles, hereby selection bias is minimised. Previous studies only included known implantation data (KID), meaning SET resulting in one or no implanted embryo or DET resulting in two or no implanted embryos [10–13, 17, 18]. An advantage of the EU-model is that it takes the chances of each specific embryo into account. Thus, it can predict if a DET will most likely result in one or two implanted embryos. Moreover, it can predict when the result is one implanted embryo, which one of the two embryos is the most likely candidate [25, 27–29]. Considering these predictions before embryo transfer, can be helpful in the decision between SET and DET. The goal is to optimize the chance for implantation, but a high probability on a twin pregnancy can be unacceptable. Discussing these considerations with patients before embryo transfer could be a valuable addition to informed- and shared decision making. However, before clinical implementation, further optimization and prospective validation of our models needs to be performed.

Second, we created additional value by including female age. Pregnancy outcome is well known to be negatively associated with female age [40]. Comparison of implantation rates between women younger than 35 years or 35 years or older, showed a significant different implantation rate for embryos with the same grade according to TLM embryo selection algorithms [21]. Our models indicate a similar distribution of pregnancy chances in relation to morphokinetic parameters, but overall chances decrease with increasing age. Our models without female age performed less than our models including female age, according to a lower AUC. However, for the purpose of individualized prediction of pregnancy chances, the inclusion of female age in the model is helpful. Without the characterization of an embryo originating from a woman of a certain age, pregnancy chances are more of an average across all female ages, resulting in a less reliable prediction for a specific couple. Insight into an age related decrease in implantation potential per embryo could also enable cost-effectiveness considerations, especially with regard to embryo selection for cryopreservation.

Furthermore, the models we developed generate continuous pregnancy chances rather than cut-off values based on specific developmental time intervals. First, no consensus is reached yet about cut-off values correlating with implantation [8, 9]. Moreover, as is the case for scoring embryos based on morphological criteria, this often results in multiple embryos with the same grade. Our models generate endless possibilities and enable a more discriminative ranking of embryos resulting from an IVF or IVF-ICSI treatment by giving each embryo an individual pregnancy chance.

The development of TLM-based embryo selection algorithms or prediction models in general remains subject to selection bias by only including data of IVF treatment outcomes of fresh embryo transfers, performed with an embryo that was selected as the best embryo by morphological evaluation. This limitation also applies to our data set. All embryo selection models require the assumption that the identified morphokinetic characteristics indicative of implantation, also apply to the rest of the embryo cohort of this patient. To our knowledge, no data exists to support or refute this assumption, as models including the treatment outcome of all transferred embryos originating from one (fresh) treatment cycle are not available. Previously published TLM embryo selection models are also based on data of fresh embryo transfer combined with morphological selection [10–14, 16–18].

A limitation of our study is that we were unable to include endocrine indicators of ovarian ageing and oocyte quality. In addition, we did not include information regarding blastocyst formation since we used morphokinetic data until day 3. A recent study showed that a low day 3 cell number was independently associated with decreased live birth rate during single blastocyst cycles [41]. This demonstrates the association between a day 3 variable and live birth and supports the use of morphokinetic parameters of the cleavage divisions for the prediction of pregnancy chances. An advantage is that the included parameters up until the 5-cell stage can be annotated easily and reliably, as evidenced by high ICC’s for inter-observer agreement found in our own study, but also others [42]. In addition, top ranking embryos can already be identified at the 5-cell stage and only cell number and morphology at 66–68 h post-fertilization needs to be determined to decide which embryo is the most likely candidate to implant. If several embryos are available with a similar high implantation potential, this knowledge can help in the decision to extend culture to the blastocyst stage.

However, our prediction models are not yet robust enough to use the calculated predictions in clinical practice. In the past, several prediction models without TLM parameters have been developed to predict pregnancy outcome, with different predictive values [43–46]. A systematic review with meta-analysis on this subject concluded that studies that focus on embryo factors that are predictive of IVF success are necessary [40]. A much larger study than ours developed a TLM embryo selection algorithm. They showed that their algorithm can predict the implantation potential of the embryos with an AUC of 0.65 [18]. The AUC’s of our prediction models without female age are remarkably lower (0.57 for model A and 0.61 for model B). During future research, we aim to apply our methods to a larger dataset and with inclusion of more TLM parameters up to the blastocyst stage to improve the predictive value. We will also explore the inclusion of more TLM parameters up to the blastocyst stage to improve the predictive value. If a satisfactory predictive value can be achieved, a well-designed prospective validation must first take place, before implementing such a TLM model in clinical practice.

Differences between IVF clinics can result in a failed external validation of embryo selection models. In our case model B performed less during external validation on data of clinic A than the other way around. We investigated the cause of this and observed the correlation between the interval t5-t4 and pregnancy to be different for model A and B. Whether this was an explanation for the lower performance during external validation of model B on clinic A, was tested by developing the models with a categorical variable for t5-t4. However, external validation of these models was not different from the original models indicating that differences in t5-t4 were not the cause of the lower performance of model B on data of clinic A. Model B was not overfit, as evidenced by internal validation. Therefore, the only remaining plausible explanation for the lower performance during external validation are procedural differences between the two clinics. This may be because of a difference in culture conditions and the fertilization method used. Indeed, throughout the study period, different culture media and oxygen rate were used. Results regarding culture media and conditions are conflicting. One study described no impact of culture medium on morphokinetics [47] while others do [48, 49]. Our developed model A is independent of fertilization method because only interval data remained in the final model; this can be an advantage for the reproducibility of the model. In model B, however, t2 remained in the final model, but here 90% of the data included ICSI treatments making differences in t2 between IVF and ICSI negligible.

## Conclusions

Our study demonstrates the use of the EU statistical model in predicting pregnancy chances according to time-lapse morphokinetics and female age. This statistical model enables the inclusion of both SET- and DET cycles irrespective of the number of implanted embryos. Hereby selection bias is minimised. Our prediction models generate continuous pregnancy chances and the addition of female age results in predictions for an individual couple. With further improvements, a potential application of our prediction models is that they can aid in the decision between SET or DET, to optimize the chance for implantation and reduce the risk of a twin pregnancy. Future research will have to clarify if our approach is able to result in a prediction model with reliable predictions to be used in clinical practice. We believe that our used methods are new in the field and add a new perspective to handling the data generated by time-lapse incubators.

## Data Availability

The data underlying this article cannot be shared publicly due to the privacy of individuals that participated in the study. The data will be shared on reasonable request to the corresponding author.

## Abbreviations

TLM: Time-lapse monitoring
SET: Single embryo transfer
DET: Double embryo transfer
IVF: in vitro Fertilization
EU statistical model: Embryo-Uterus statistical model
AUC: Area under the curve
CI: Confidence interval
FSH: Follicle stimulating hormone
hCG: Human chorionic gonadotropin
ICSI: Intracytoplasmic sperm injection
tPNa: Time of pronuclear appearance
tPNf: Time of pronuclear fading
t2: Time of reaching the 2-cell stage
t3: Time of reaching the 3-cell stage
t4: Time of reaching the 4-cell stage
t5: Time of reaching the 5-cell stage
t6: Time of reaching the 6-cell stage
t7: Time of reaching the 7-cell stage
t8: Time of reaching the 8-cell stage
ICC: Intra-class correlation coefficient
KID: Known implantation data
